# Pathways to Sustainable Health Care Development: Study on the Carbon Reduction Potential of Telemedicine in China

**DOI:** 10.2196/63927

**Published:** 2025-02-24

**Authors:** Zhi Li, Lianrui Xiang, Jing Ning, Wenbo Li, Yong Huang, Xue Xiao

**Affiliations:** 1 Public Service and Development Office, Key Laboratory of Birth Defects and Related Diseases of Women and Children West China Second University Hospital Sichuan University Chengdu China

**Keywords:** telemedicine, carbon reduction potential, carbon emissions, energy consumption, sustainable development

## Abstract

**Background:**

Carbon emissions are a global concern due to their significant greenhouse effect. The health care sector’s greenhouse gas (GHG) emissions must be controlled. Telemedicine in China continued to grow between 2020 and 2022, offering a promising solution for reducing carbon emissions in the country’s health sector.

**Objective:**

This study explores the potential of telemedicine for reducing GHG emissions and saving energy through a life cycle assessment.

**Methods:**

This study used the Chinese Environmentally Extended Input-Output database, which is based on input-output models, to quantify the GHG emissions and energy consumption associated with care outpatient and telemedicine from a life cycle perspective. Data collected from the West China Second University Hospital of Sichuan University between 2020 and 2022 were incorporated into the analyses.

**Results:**

The findings indicated that telemedicine could reduce GHG emissions by 36 tCO_2_e. The GHG emissions per telemedicine session were only 19.14 kgCO_2_e and are expected to decrease from 2025 to 2030. Replacing in-person treatments with telemedicine can lead to an average reduction of 85.51 kgCO_2_e emissions.

**Conclusions:**

In the future, the widespread adoption of telemedicine could help achieve carbon neutrality in the health care sector. Telemedicine is crucial for establishing the sustainable development of the health care sector.

## Introduction

### Background

Excessive greenhouse gas (GHG) emissions and energy usage have garnered global attention as the greenhouse impact intensifies. The Paris Agreement mandates a 43% reduction in GHG emissions by 2030 to achieve net zero by 2050 [1]. The health care sector’s GHG emissions are 1.57 times higher than those of the worldwide air transport industry, estimated at 4.4% of total global net emissions, with the United States, China, and European Union countries responsible for 56% of the total global health care GHG emissions. China’s health care sector is the third largest contributor to carbon emissions globally [2]. As one of the leading nations in global carbon emissions, China has been implementing measures to conserve energy and diminish carbon output. China is aiming to achieve net zero carbon emissions by promoting scientific and technological advancements and developing a comprehensive low-carbon technological innovation framework [3]. A correlation exists between expenditure in the industrial sector and GHG emissions. The total expenditure of China’s health care sector as a proportion of the gross domestic product (GDP) increased from 6.43% in 2018 to 7.12% in 2020 [4]. A disparity persists between China’s health care expenditures and those of industrialized nations. As the health care industry continues to expand, its GHG emissions will also increase. The health care sector in China must use multiple strategies to mitigate GHG emissions and conserve energy.

Telemedicine refers to the use of network and information and communication technologies (ICTs) to provide remote access to health care services. It is widely used to deliver telehealth care and reduce medical expenses for patients [5,6]. The evolution of telemedicine in China has transpired via 3 phases. The initial phase, spanning from 2000 to 2015, was the early exploration period, giving rise to medical information and medical consultation businesses. The second phase, spanning from 2016 to 2019, was known as the policy development period, during which telemedicine-related policies were progressively implemented, enabling the integration of telemedicine into diagnostics. The third phase, from 2020 to 2022, was characterized by remarkable business expansion. In the context of the COVID-19 pandemic, in-person outpatient visits were halted, leading to an unprecedented surge in the demand for telemedicine. This shift altered public perceptions of health care, prompting numerous public medical institutions to adopt telemedicine, thereby enhancing its significance.

### Literature Review

Telemedicine has cost-reduction benefits for patients who require time off from work, undergo alternative treatment, or face long-distance transportation challenges [7,8]. It can improve patients’ access to health care by reducing the overall trip time and distance, making it more convenient for patients to receive in-person treatment [9]. The current clinical implementation of telemedicine in China primarily focuses on managing chronic diseases in internal medicine and providing home care services for postoperative patients. By enabling virtual communication, telemedicine can enhance patient care and satisfaction [10-12].

Previous research has demonstrated that telemedicine has beneficial environmental effects. It is acknowledged as a viable approach to reduce the carbon footprint by eliminating the need for patients to travel to health care facilities, potentially decreasing GHG emissions. Moreover, it can provide financial savings for patients in rural areas while promoting environmental sustainability [13,14]. Several studies have examined carbon emissions associated with telemedicine services in some regions. The findings suggest that teleconsultations delivered via telephone and video platforms are more environmentally sustainable than in-person consultations [15-17].

Telemedicine has grown significantly since the 21st century and gained widespread adoption, particularly during the COVID-19 pandemic in China. Research in telemedicine has primarily focused on system construction, remote consultation technology, and clinical application. While China’s telemedicine policies are based on overarching regulations, they lack supportive measures such as financial assistance and proactive promotion [18]. To ensure the sustainable advancement of telemedicine, the government must increase its investment and construct a long-term operational framework [19]. Implementing telemedicine access policies will impact the construction and sustainable development of telemedicine platforms [20]. As a low-carbon development approach, telemedicine strategies should be tailored to local infrastructure conditions [21]. Artificial intelligence, blockchain, and robotics present new opportunities for the development of telemedicine, enabling direct interactions between doctors and remote patients through various platform models [22]. With advancements in medical service policies and telemedicine technology, China’s ecological welfare performance and citizen-centered sustainable community services will continue to improve [23,24].

Prior studies have not conducted quantitative analyses on the ecological impacts and sustainable development of telemedicine in China. Given its extensive applicability, the steady development of telemedicine is important. This study aims to examine the benefits of telemedicine in reducing GHG emissions and conserving energy while also forecasting GHG emissions from outpatient visits and telemedicine utilization in China.

A model was developed using the Chinese Environmentally Extended Input-Output (CEEIO) database within the framework of life cycle assessment (LCA). LCA is an approach used to assess the environmental effects of products, processes, or activities through their life cycle, encompassing raw material acquisition, product manufacturing, transportation, distribution, utilization, potential reuse, maintenance, and ultimate disposal [25]. The LCA model has been applied to assess GHG emissions across various sectors in China, including energy, nuclear power, urban transportation systems, and biofuels [26-28]. As a comprehensive approach, LCA is also applicable for calculating energy conservation and emission reductions in telemedicine services within medical institutions. The CEEIO database has previously been used to conduct environmental assessments in China’s building sector, quantify the environmental impact of resource extraction in China, and conduct input-output analyses of China’s home water footprint [29-31].

Some studies have quantified the carbon emissions of telemedicine in medical institutions. In 2021, telemedicine services contributed to a 17,000 metric ton reduction in GHG emissions by Stanford Health Care [17]. An investigation of 17 research institutes in Italy indicated that a single telemedicine consultation might reduce carbon emissions by 13 kg [16]. Additionally, face-to-face outpatient clinics at the University Hospital of Northern Sweden generated 40 to 70 times more carbon emissions than telemedicine [15]. Thus, telemedicine has greater environmental advantages.

Despite extensive research on telemedicine policies and clinical applications, with some countries examining its low-carbon potential as an innovative technology, there is a lack of studies on telemedicine’s contribution to sustainable development and its life cycle carbon emissions in China. As China pursues carbon neutrality and prioritizes a people-centric development strategy, enhancing the governmental recognition of the low-carbon characteristics of telemedicine is crucial for the sustainable advancement of China’s health sector.

This study estimates the GHG emissions and energy consumption associated with telemedicine in China, evaluating its environmental advantages by developing a life cycle model based on patient-related data from teleconsultations at the West China Second University Hospital of Sichuan University (WCSUH-SCU) from 2020 to 2022. WCSUH-SCU, directly supervised by the National Health Commission of China, is one of the top national tertiary hospitals and serves as a medical center for women and children in southwest China, playing a vital role in medical service, education, and research.

This study aims to reveal the level of telemedicine development and its associated ecological sustainability in southwest China. Consequently, it provides essential data to bolster the sustainable advancement of China’s medical sector and offers policy recommendations for decision makers.

## Methods

### Research Boundaries

The research boundary of this study spans the entire process from beginning to end. At WCSUH-SCU, the telemedicine research boundary covers the development, establishment, and utilization of the telemedicine platform. Similarly, the research boundary on outpatient services at WCSUH-SCU extends from establishing the outpatient appointment platform to the in-person consultations.

**Figure 1:**
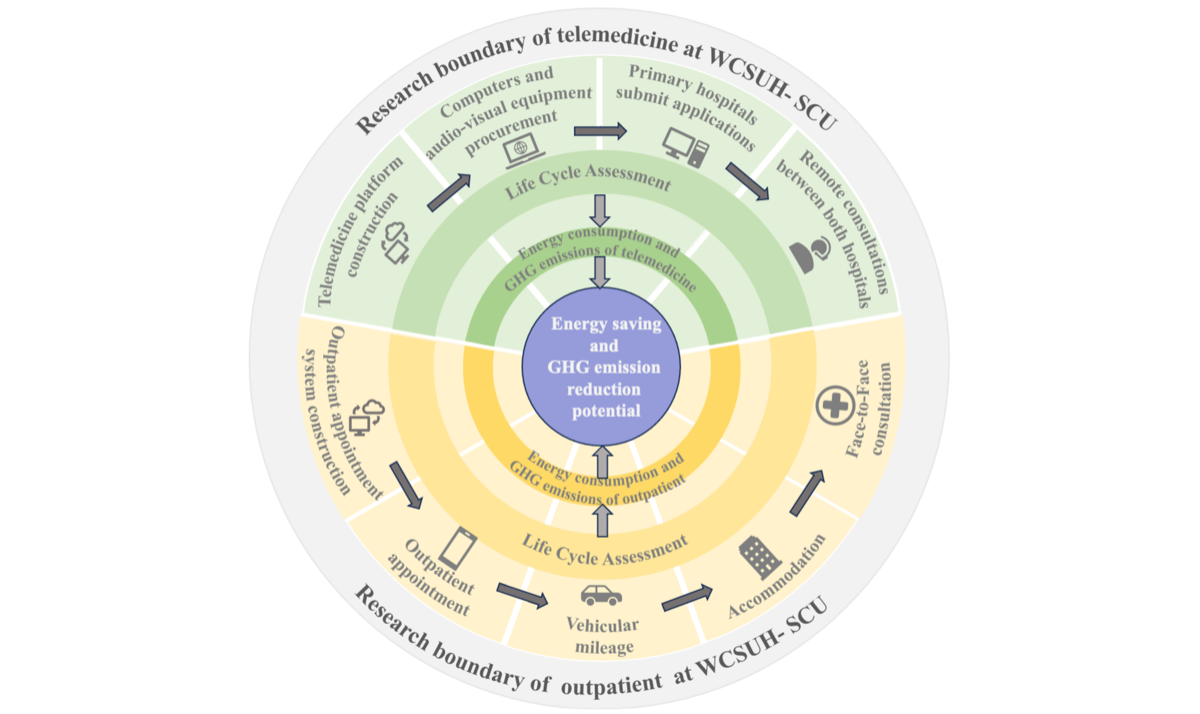
The research boundaries for telemedicine and outpatient of the West China Second University Hospital of Sichuan University (WCSUH-SCU). GHG: greenhouse gas.

The primary focus of this study is to explore the environmental benefits of telemedicine by estimating GHG emissions within both outpatient and telemedicine boundaries for the same patient. As shown in Figure 1, the outpatient appointment stage mainly includes developing the outpatient appointment system and scheduling outpatients. During the treatment stage, patients visit WCSUH-SCU for in-person consultations, with transportation assumed to be via passenger cars. Telemedicine life cycle management mainly includes the construction and usage of telemedicine platforms. The construction stage involves establishing an online telemedicine platform and purchasing computers and audiovisual equipment. During the utilizing stage, all processes are handled by the hospital administrators and doctors through computers and audiovisual equipment.

### Study Design

LCA summarizes and evaluates the energy and resource inputs and outputs of a product or activity throughout its life cycle, along with its potential environmental impact. In this study, we use LCA to evaluate the GHG emissions and energy consumption of telemedicine and outpatient services in China. Input-output models, commonly used in LCA, estimate embodied energy and carbon emissions based on industrial sector expenditures.

Previous health care environmental impact studies have used similar methods to estimate emissions from preoperative evaluation and cataract surgery [32,33]. In this study, the CEEIO database was used to quantify the GHG emissions from outpatient and telemedicine services. Additionally, Ecoinvent (version 3.9; Ecoinvent Association), a background database in LCA and other environmental assessments, was used to estimate the energy consumption per unit mileage of the car throughout its life cycle. A detailed calculation for the GHG emission reduction and energy-saving benefits of telemedicine is introduced as follows.

The CEEIO database is the result of extensive research based on the environmental extended input-output model tailored to the Chinese context. It consists of benchmark economic input-output tables published by China’s government statistical agencies and statistical data from multiple sources, enabling an extensive analysis of the environmental footprint of various industries in China. In this study, the GHG and energy intensities of China’s industries were obtained from version 3.2 of the CEEIO database [34]. Detailed data on these GHG and energy intensities can be found in Table 1, where the energy consumption intensity of the software and health services is derived from the conversion of CO_2_e emissions per unit standard carbon.

**Table 1:** GHG^a^ intensities for outpatient and telemedicine industries.

Industry	GHG intensity (gCO_2_e^b^/RMB^c^)	Energy intensities (gce^d^/RMB)
Software services	2.66	1.08
Computer	0.72	2.05
Audiovisual equipment	0.72	2.05
Hotels	4.33	4.44
Health services	2.66	1.08

^a^GHG: greenhouse gas.

^b^The CO_2_e emission per gram of standard coal is 2.46 g

^c^RMB: Renminbi (official Chinese currency).

^d^gce: grams of standard coal equivalent.

The GHG intensities due to transportation, calculated as the weighted average of passenger car emissions in China from 2020 to 2022, were 212.2, 236.07, and 247.77 g CO_2_e/km, respectively [35-37]. According to the LCA from version 3.9 of Ecoinvent, the energy consumption per unit mileage of passenger cars was estimated to be 3.95 MJ/km. For electricity, the GHG intensities from 2020 to 2022 were 0.6101, 0.581, and 0.5703 kgCO_2_e/kWh, respectively [38,39], while energy intensities were 0.305, 0.303, and 0.302 kgCO_2_e/kWh, respectively [40].

This study used relevant data from patients who underwent telemedicine treatment over a specific period as samples, combined with economic data to calculate the GHG emissions of outpatient service and telemedicine under the same sample. This approach helped confirm the advantages of telemedicine for energy savings and GHG emission reduction.

A mathematical model was developed to estimate the GHG emission and energy consumption of both outpatient and telemedicine services, allowing for an assessment of telemedicine’s GHG emission reduction and energy-saving benefits. The details of the method are described by the following 3 equations:



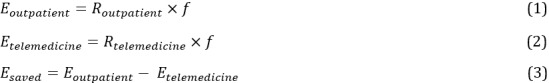



where *E_outpatient_* and *E_telemedicine_* represent the GHG emissions and energy consumption from outpatient and telemedicine services, respectively. *R_outpatient_* and *R_telemedicine_* represent the revenue mobilized by each industry involved in outpatient and telemedicine services, respectively. R represents the transportation distance and duration of telemedicine. Similarly, *f* not only represents the GHG and energy intensities of each industry involved in outpatient and telemedicine services, respectively, but also the GHG emissions and energy consumption per unit mileage of transportation, electric GHG emission factors, and energy consumption factors of electricity. *E_saved_* represents the GHG emission reduction and energy saving of telemedicine.

### Data Collection

The study sample comprised relevant data from 421 patients who completed telemedicine treatment between 2020 and 2022. The data set included the patient’s primary hospital, telemedicine department, duration of telemedicine treatment, and telemedicine and outpatient service fees charged by participating doctors. Actual values are displayed in Tables S1 and S2 in Multimedia Appendix 1, taking the telemedicine data from WCSUH-SCU in 2020 as an example.

The round-trip distance for outpatient visits was calculated using the optimal driving route from the primary hospital to WCSUH-SCU, modeled through Python (version 3.7; Python Software Foundation) with Baidu Maps. Actual transportation distances for patients traveling to WCSUH-SCU for outpatient services are shown in Table S3 in Multimedia Appendix 1, using 2020 as a reference.

A hotel reservation was deemed necessary for trips exceeding 300 kilometers one way. Hotel prices were derived from the quarterly average prices of star-rated hotels in Chengdu from 2020 to 2022 [41]. The development costs of the telemedicine platform and outpatient appointment system were derived from the costs incurred by software companies collaborating with WCSUH-SCU. Outpatient and telemedicine service fees were determined according to the standards set by the Sichuan Provincial Development and Reform Commission and Sichuan Provincial Health Care Security Administration [42,43].

### Data Analysis

A descriptive analysis was conducted on the distribution of telemedicine departments within WCSUH-SCU. Through modeling and data analysis, determinants influencing GHG emissions and energy consumption in outpatient and telemedicine were prioritized to reveal the principal causes impacting the GHG emissions and energy consumption in outpatient and telemedicine services.

According to the existing literature, patient travel and electricity are the main factors affecting the sustainability of the health sector. This study constructed predictive models based on the average GHG emissions from transportation unit mileage, and electric GHG emission factors were established to reveal the future sustainable development of China's health care sector. The average GHG emissions related to transportation unit mileage from 2023 to 2030 were calculated by weighting the GHG emissions unit mileage of China’s passenger cars from 2023 to 2030 with forecast data on vehicle ownership. Carbon emissions per unit mileage of China’s passenger cars from 2023 to 2030 were based on forecast data obtained from the China Automobile Low Carbon Action Plan 2021 and 2022, including information on the carbon emissions of various energy vehicles in China. Additionally, forecast data on vehicle ownership with different energy sources in China from 2023 to 2030 were sourced from an automotive aftermarket white paper published by Roland Berger in 2022 [44]. Electric unit GHG emissions were calculated through variations in the proportion of nonfossil energy power generation combined with electricity intensities. The 14th Five-Year Plan for a Modern Energy System anticipates that the proportion of nonfossil energy in power generation will reach 39% by 2025. Simultaneously, the 2023 China Electric Power Industry Annual Development Report anticipates that the share of nonfossil energy power generation will reach 50% by 2030 [40].

## Results

### Descriptive Analyses

**Figure 2:**
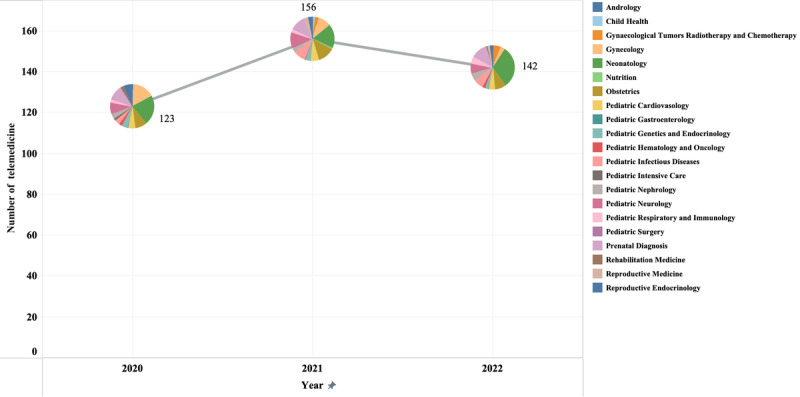
Distribution of telemedicine implementation departments in West China Second University Hospital of Sichuan University.

Between 2020 and 2022, a cumulative total of 421 telemedicine consultations were documented. The distribution of teleconsultation departments of WCSUH-SCU from 2020 to 2022 is shown in Figure 2. Over 3 years, telemedicine was implemented throughout 19 distinct clinical departments, encompassing reproductive medicine, pediatric subspecialties, obstetrics, and gynecology. The consultations predominantly comprised pediatric subspecialties, increasing from 55.3% (68/123) in 2020 to 65.5% (93/142) in 2022. The proportion of telemedicine in obstetrics and gynecology declined from 25.2% (31/123) in 2020 to 11.3% (16/142) in 2022. Since the onset of the COVID-19 pandemic in early 2020, the incidence of coronavirus infections among children in China has risen markedly. On the other hand, the volume of gynecological surgery patients has persistently declined due to the challenges associated with in-person medical care for epidemic control. By 2022, the prevalence of telemedicine in neonatology and prenatal diagnosis rose to 31% (44/142) and 12% (17/142), respectively, diverging from the declining trend of China's birth population from 2020 to 2022, primarily due to the Chinese government's enhancement of prenatal screening for expectant mothers and newborn disease assessments.

### Reduction in GHG Emission and Energy Saving Through Telemedicine

**Figure 3:**
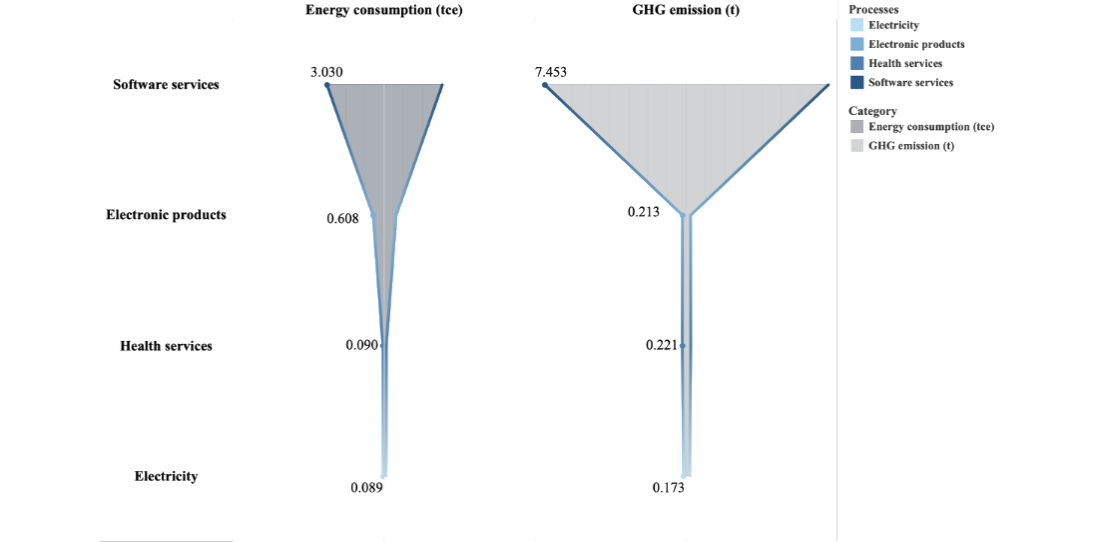
Energy consumption and greenhouse gas (GHG) emissions due to telemedicine services.

Between 2020 and 2022, telemedicine services at WCSUH-SCU reduced GHG emissions by 36 tCO_2_e, and their total life cycle emissions amounted to 8.06 tCO_2_e, as shown in Figure 3. According to the life cycle management, the GHG emissions by telemedicine per patient amounted to 19.14 kgCO_2_e. The software design and programming stage significantly contributed to telemedicine’s GHG emissions, totaling 7.45 tCO_2_e and representing 92.5% of the total. The amount of GHG emitted due to telemedicine use was 0.39 tCO_2_e. Between 2020 and 2022, the energy-saving potential of telemedicine at WCSUH-SCU was 22.58 tce, while the total energy usage for telemedicine during this period was 3.82 tce. The energy consumption of software design and programming was measured at 3.03 tce, representing 79.4% of the energy consumed by telemedicine, thus remaining the largest contributor to energy consumption in this field. Furthermore, as energy consumption rose to 15.9%, electronic products, including computers and video equipment, emerged as the second largest contributor, consuming 0.61 tce.

**Figure 4:**
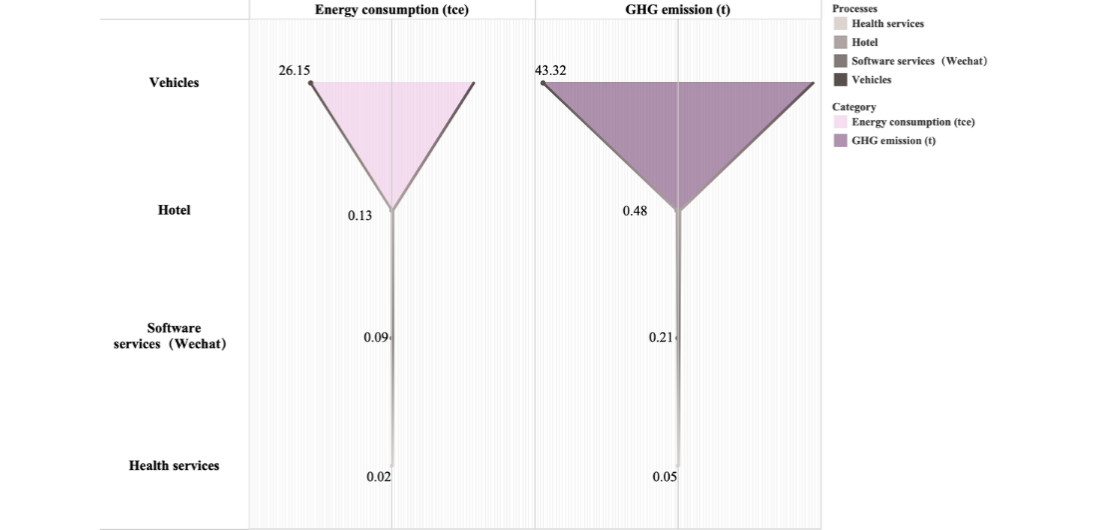
Energy consumption and greenhouse gas (GHG) emissions due to outpatient services.

As illustrated in Figure 4, cumulative GHG emissions from outpatients between 2020 and 2022 totaled 44.06 tCO_2_e, with per capita GHG emissions for outpatients reaching 104.66 kgCO_2_e. Outpatient GHG emissions were higher than those of telemedicine services. Transportation-related GHG emissions significantly contributed to outpatient emissions, totaling 43.32 tCO_2_e, which represented 98.3% of the overall emissions. Between 2020 and 2022, the total energy consumption due to outpatient services was 26.39 tce, 6.9 times greater than that of telemedicine. The energy consumption by vehicles remained the largest contributor at 26.15 tce, representing 99.1% of the overall energy consumption of outpatient services.

Thus, telemedicine demonstrates a clear advantage in reducing emissions and conserving energy. This indicates that the effective implementation of telemedicine has the potential to improve health sector sustainability in China by enhancing energy conservation and reducing GHG emissions.

Regarding the transportation methods employed in this study, the development of clean energy and the increase in the market share of new energy vehicles are expected to help reduce GHG emissions related to transportation. In turn, this reduction is anticipated to have a positive impact on outpatient emissions. Moreover, optimizing China’s energy-producing system, including enhancing hydropower and wind power, will lead to a simultaneous decrease in electric GHG emissions. Consequently, telemedicine will arise as a more environmentally friendly option for health care in China. An estimation of future GHG emissions from transportation and electricity will then be projected based on the present policy.

### Scenario Analysis

#### Key Factors of GHG emissions

To better demonstrate telemedicine’s low-carbon characteristics and provide data to support its application and promotion, this study forecasted GHG emissions for both telemedicine and outpatient care from 2023 to 2030 based on key emission factors.

Between 2020 and 2022, transportation accounted for an average of 98.5% of GHG emissions during the patient treatment stage, making it the primary contributor to outpatient GHG emissions. Given that electricity is essential for telemedicine, it is commonly acknowledged as the main driver of telemedicine’s GHG emissions.

In contrast, between 2020 and 2022, electricity was the main source of telemedicine-related emissions, averaging 75.2%. These findings highlight the significant role of electricity in contributing to GHG emissions. The primary determinants of outpatient and telemedicine GHG emissions in this study align with the findings of prior research. The GHG emissions of essential components significantly impact outpatient and telemedicine. Variations in GHG emissions from outpatient and telemedicine could be reflected in the variations in GHG emissions from transportation and electricity.

This study employed scenario analysis to forecast GHG emissions during medical treatment for patients, including telemedicine use. GHG emissions from transportation unit mileage and electricity from 2023 to 2030 were assessed according to existing national carbon emission policies. The projected GHG emissions for transportation unit mileage and electric unit GHG emissions from 2023 to 2030 are shown in Table 2.

**Table 2:** Projected average GHG^a^ emissions for transportation per unit mileage and electricity.

Year	Transportation unit mileage GHG emissions (g CO_2_e/km)	Electric unit GHG emissions (kgCO_2_e/kWh)
2022	247.770	0.570
2023	254.419	0.563
2024	248.739	0.556
2025	242.776	0.549
2026	235.205	0.533
2027	227.208	0.516
2028	218.786	0.500
2029	208.402	0.483
2030	195.843	0.467

^a^GHG: greenhouse gas.

#### Predictive Results of GHG Emissions

**Figure 5:**
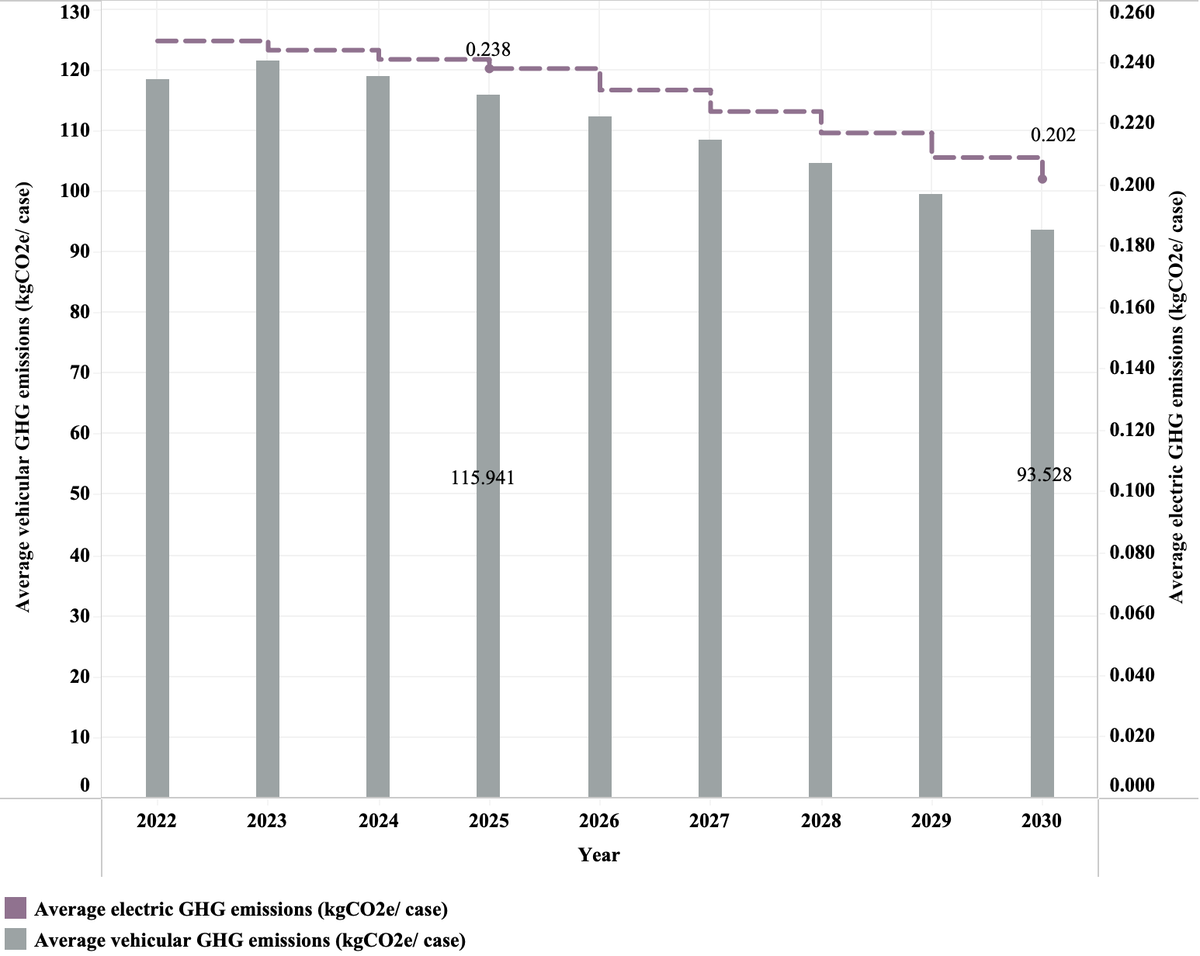
The predictive results of greenhouse gas (GHG) emissions for transportation and electricity.

Using the forecast results and 2022 teleconsultation data from WCSUH-SCU, the average transportation and electricity-related GHG emissions from 2023 to 2030 were estimated. As shown in Figure 5, there will be a brief increase in the average transportation-related GHG emissions from 2020 to 2024. The average transportation-related GHG emissions in 2025 were estimated to decrease by 2% compared to the corresponding emissions in 2022, reaching 115.94 kgCO_2_e per case. This downward trend was expected to continue, with emissions expected to drop to 93.528 kgCO_2_e per case in 2030. It is evident that with the increasing adoption of new energy vehicles, there will be a significant reduction in average transportation-related GHG emissions after 2025 compared to the preceding period.

Regarding electricity-related GHG emissions, a 3.7% reduction was projected by 2025 compared to 2022, reaching 0.238 kgCO_2_e per case. Furthermore, the average electricity-related GHG emissions in 2030 will reduce by 15% compared to 2022. Implementing power generation structural reform has contributed to low-carbon development within the power generation industry.

Since GHG emissions from transportation account for a huge proportion of outpatient GHG emissions, GHG emissions from an average outpatient visit are expected to continue to decrease from 2025 to 2030. Depending on the effect of alterations in the average electricity-related GHG emissions from 2022 to 2030, it is anticipated that the GHG emissions of average telemedicine usage will also continue to decline from 2025 to 2030.

## Discussion

### Principal Results and Contributions

This study concludes that telemedicine may significantly decrease GHG emissions and energy consumption compared to in-person outpatient consultations, as determined by the LCA. Telemedicine usage leads to an average decrease of 85.51 kgCO_2_e emissions. In 2022, the total number of telemedicine consultations in China was 26.7 million, potentially reducing CO_2_e emissions by a total of 2,283,117 metric tons. This corresponds to the yearly carbon emissions of 280,826 individuals in China [45]. According to the Mortality Cost of Carbon Model, the reduction in GHG emissions due to telemedicine could have saved 515 lives from climate change in 2022 [46]. The widespread implementation of telemedicine can markedly reduce GHG emissions and mortality rates. Telemedicine can serve as an excellent strategy for promoting environmental sustainability, reducing GHG emissions, and enhancing human health [47].

The health care sector safeguards both public health and environmental health. Expanding the accessibility and prevalence of telemedicine while minimizing carbon emissions from patient travel to hospitals is an efficient strategy to attain China’s carbon neutrality objective in the health care sector [2]. Compared to the operational carbon emissions of medical institutions, telemedicine offers a clear advantage in reducing carbon emissions. This study, consistent with prior research, suggests that computers, audiovisual equipment, web servers, and electricity costs contribute to telemedicine-related GHG emissions [7]. As conventional manufacturing and technology companies globally implement low-carbon strategies to mitigate their energy consumption, there is a progressive decline in carbon emissions across the life cycle of technology-related equipment, enhancing telemedicine’s sustainability. Concurrently, sustainable energy serves as an additional impetus for the low carbonization in telemedicine. China's clean energy consumption is anticipated to rise from 25.9% in 2023 to 60% by 2030, making telemedicine a more environmentally sustainable technology [40]. As climate change intensifies, telemedicine’s sustainable development of telemedicine is essential for the health care sector.

Transportation emissions remain the largest contributor to GHG emissions in outpatient care [48]. Telemedicine eliminates the need for patient travel, thereby reducing GHG emissions. China's major hospitals are predominantly concentrated in economically prosperous urban areas. However, mountainous regions in China account for 64.9% of the country's total land area, housing approximately 330 million permanent residents [49]. Patients living in mountainous regions emit large amounts of GHG when traveling to hospitals. Therefore, telemedicine can be a key solution for reducing travel-related GHG emissions.

This study confirms that in-person treatments generate more GHG than telemedicine. As 5G communication technologies and artificial intelligence continue to expand telemedicine’s capabilities, more in-person treatments can be replaced. In 2022, the aggregate number of medical treatments in China was 8.42 billion, signifying a considerable potential for GHG emission reduction if telemedicine were to supplant in-person treatments.

To support the construction of a high-quality ecological environment, China has promulgated the Technology-Supported Carbon Peak Carbon Neutrality Implementation Plan (2022-2030), which proposes to achieve green development of hospital management based on green and low-carbon technological innovation. Telemedicine is innovative compared to traditional outpatient clinics. Furthermore, with the development of internet technology, more advantages and potential uses of telemedicine are coming to light. Large hospitals can make full use of telemedicine to help achieve hierarchical diagnosis and treatment. As China’s medical industry shifts from a focus on medical insurance services to health services, telemedicine will expand beyond hospital diagnosis and treatment to include health monitoring and management of healthy individuals. As an innovative low-carbon technology, telemedicine will contribute significantly to the reduction of carbon emissions in China’s health care industry.

Climate change is a global health crisis, and the health care sector—one of the fastest-growing sectors in terms of GDP—must take responsibility for reducing carbon emissions. Without effective intervention, global health care emissions could rise to 6 billion tons by 2050 [2]. The health care sector must promote decarbonization by developing sustainable solutions to reduce carbon emissions and improve human health. Telemedicine, as a win-win solution, should be taken seriously by the public. Despite the increased use of telemedicine during the COVID-19 epidemic, there is a lack of public awareness regarding its economic and environmental benefits. Government officials must enhance their comprehension of telemedicine’s low emission advantages and implement effective procedures to facilitate its widespread adoption.

### Limitations

This study uses an LCA to explore the potential of telemedicine to reduce GHG emissions and save energy. However, there are some limitations. First, transportation-related GHG emissions and energy consumption were estimated based on passenger cars. However, the diversity of transportation modes should be considered. Second, GHG emissions and energy consumption from other fixed assets, such as furniture, air conditioners in the consultation space, and network transmission during the consultation, were not included in this GHG emission reduction and energy-saving estimation. In future work, more factors affecting telemedicine carbon emissions should be included in the estimation. Third, in the carbon emission prediction section, we estimated future GHG emissions for outpatient and telemedicine services based on transportation and electricity usage without considering other factors. In future studies, more complex prediction models should be developed to predict telemedicine-related GHG emissions.

### Conclusion

This study evaluates the environmental impact of telemedicine compared to traditional outpatient modes using a life cycle–based mathematical model. The findings confirm that telemedicine significantly reduces GHG emissions. From 2020 to 2022, 421 teleconsultations resulted in a total reduction of 36 tCO_2_e in GHG emissions. Each telemedicine visit resulted in only 19.14 kgCO_2_e of GHG emissions, reducing emissions by 85.51 kgCO_2_e compared to in-person visits. Additionally, telemedicine use resulted in an average savings of 53.63 kg of standard coal. The primary contributors to life cycle GHG emissions in outpatient and telemedicine services were transportation and system programming design. Scenario analysis predicted that GHG emissions from telemedicine and outpatient services will continue to decline from 2025 to 2030, contributing to an overall decline in health care sector GHG emissions.

In 2022, China recorded 26.7 million telemedicine consultations, resulting in a potential reduction of 2,283,117 tons of CO_2_e emissions. This reduction is equivalent to the annual carbon emissions of 280,826 individuals in China and could save 515 lives from climate change. Telemedicine serves as a dual-benefit measure by enhancing the efficiency of medical services and promoting the sustainable development of the health care sector. As the proportion of clean energy increases and ICT advances, telemedicine can be applied across more medical fields, thereby contributing more significantly to the health care sector's carbon neutrality efforts. Moreover, as the share of health services expands, telemedicine will extend beyond hospital diagnosis and treatment to encompass health monitoring and management for healthy individuals. Government decision-makers should deepen their comprehension of telemedicine's low-emission advantages and formulate effective legislation to facilitate its broad implementation.

In conclusion, this study quantifies telemedicine’s role in reducing carbon emissions in China’s health sector, emphasizing its significance in achieving carbon neutrality. It not only presents factual evidence to substantiate the sustainable advancement of China’s health sector but also offers a theoretical foundation for the future eco-friendly development of China’s medical industry. While conducted within a Chinese context, the conclusions can extend to other nations pursuing similar sustainability goals.

## Appendix

Multimedia Appendix 1 The actual data of telemedicine at West China Second University Hospital of Sichuan University in 2020.

## Abbreviations

CEEIO: Chinese Environmentally Extended Input-Output
GDP: gross domestic product
GHG: greenhouse gas
LCA: life cycle assessment
WCSUH-SCU: West China Second University Hospital of Sichuan University

## Data Availability

All data generated or analyzed during this study are included in this published article and its supplementary information files.
