# Supplementary material for: Pathways to Sustainable Health Care Development: Study on the Carbon Reduction Potential of Telemedicine in China
Source: J Med Internet Res. 2025 Feb 24;27:e63927. doi: 10.2196/63927 (PMC11894342; doi:10.2196/63927)
Supplement: Multimedia Appendix 1 [file jmir_v27i1e63927_app1.docx]

**Supplementary Material**

| Primary hospitals applying for telemedicine | Number of telemedicine |
| --- | --- |
| China Five Metallurgical Group Co., LTD. Hospital | 10 |
| Chengdu Qingbaijiang Maternal and Child Health Care Hospital | 1 |
| Deyang Jingyang Maternal and Child Health Hospital | 2 |
| Maternal and Child Health Hospital of Dujiangyan | 1 |
| Maternal and Child Health Hospital of E Mei | 3 |
| Ganzi Tibetan Autonomous Prefecture People's Hospital | 23 |
| Maternal and Child Health Hospital of Guangyuan | 1 |
| Guangyuan Central Hospital | 16 |
| HanYuan People's Hospital | 5 |
| Mianyang Youxian Maternal and Child Health Hospital | 7 |
| Mianzhu Maternal and Child Health Care Hospital | 5 |
| The People's Hospital of Ningnan County | 1 |
| Panzhihua Maternal and Child Health Hospital | 5 |
| Chongqing Rongchang Maternal and Child Health Hospital | 4 |
| Shifang Maternal and Child Health Hospital | 5 |
| The First People's Hospital of Shuangliu District, Chengdu | 5 |
| Chengdu Shuangliu District Maternal and Child Health Hospital | 7 |
| Xindu District People's Hospital of Chengdu | 8 |
| Yingjing County People's Hospital | 4 |
| Yibin Second People's Hospital | 1 |
| Zhaotong Second People's Hospital | 9 |

**Table S.1.** The actual value of telemedicine at WCSUH-SCU in 2020

**Table S.2.** The actual disease types of telemedicine at WCSUH-SCU in 2020

| Disease types of telemedicine | Number of telemedicine |
| --- | --- |
| Gynecology | 20 |
| Obstetrics | 11 |
| Prenatal Diagnosis | 12 |
| Rehabilitation Medicine | 3 |
| Pediatric Intensive Care | 2 |
| Andrology | 1 |
| Reproductive Endocrinology | 8 |
| Pediatric Infectious Diseases | 5 |
| Pediatric Respiratory and Immunology | 3 |
| Pediatric Genetics and Endocrinology | 5 |
| Pediatric Neurology | 11 |
| Pediatric Nephrology | 4 |
| Pediatric Surgery | 1 |
| Pediatric Gastroenterology | 1 |
| Pediatric Cardiovasology | 6 |
| Pediatric Hematology and Oncology | 3 |
| Neonatology | 27 |
